# Long‐term spinal cord stimulation modifies canine intrinsic cardiac neuronal properties and ganglionic transmission during high‐frequency repetitive activation

**DOI:** 10.14814/phy2.12855

**Published:** 2016-07-12

**Authors:** Frank M. Smith, Michel Vermeulen, René Cardinal

**Affiliations:** ^1^ Department of Medical NeuroscienceFaculty of MedicineDalhousie UniversityHalifaxNova ScotiaCanada; ^2^ Department of PharmacologyFaculté de médecineUniversité de Montréal and Centre de rechercheHôpital du Sacré‐Cœur de MontréalMontréalQuebecCanada

**Keywords:** Atropine, intrinsic cardiac neurones, neuronal excitability, spinal cord stimulation, synaptic transmission

## Abstract

Long‐term spinal cord stimulation (SCS) applied to cranial thoracic SC segments exerts antiarrhythmic and cardioprotective actions in the canine heart in situ. We hypothesized that remodeling of intrinsic cardiac neuronal and synaptic properties occur in canines subjected to long‐term SCS, specifically that synaptic efficacy may be preferentially facilitated at high presynaptic nerve stimulation frequencies. Animals subjected to continuous SCS for 5–8 weeks (long‐term SCS:* n* = 17) or for 1 h (acute SCS:* n* = 4) were compared with corresponding control animals (long‐term: *n* = 15, acute: *n* = 4). At termination, animals were anesthetized, the heart was excised and neurones from the right atrial ganglionated plexus were identified and studied in vitro using standard intracellular microelectrode technique. Main findings were as follows: (1) a significant reduction in whole cell membrane input resistance and acceleration of the course of AHP decay identified among phasic neurones from long‐term SCS compared with controls, (2) significantly more robust synaptic transmission to rundown in long‐term SCS during high‐frequency (10–40 Hz) presynaptic nerve stimulation while recording from either phasic or accommodating postsynaptic neurones; this was associated with significantly greater posttrain excitatory postsynaptic potential (EPSP) numbers in long‐term SCS than control, and (3) synaptic efficacy was significantly decreased by atropine in both groups. Such changes did not occur in acute SCS. In conclusion, modification of intrinsic cardiac neuronal properties and facilitation of synaptic transmission at high stimulation frequency in long‐term SCS could improve physiologically modulated vagal inputs to the heart.

## Introduction

Spinal cord stimulation (SCS) applied to upper thoracic SC segments is a neuromodulatory approach used for the treatment of refractory angina pectoris (Eliasson et al. 1996; Ekre et al. 2002). Experimental data from canine studies suggest that long‐term SCS holds some promise for the control of atrial tachyarrhythmias (Bernstein et al. 2012; Ardell et al. 2014) and to improve ventricular function in heart failure (Lopshire et al. 2009), conditions in which cardiac vagal influences (Cardinal et al. 2010) and intracardiac ganglionic neurotransmission (Bibevski and Dunlap 1999) are dysregulated. The usefulness of SCS for treating congestive heart failure is currently under clinical investigation (Singh et al. 2014; Buckley et al. 2015; Tse et al. 2015). However, the main application for SCS is the treatment of chronic pain and peripheral vascular disease (Wu et al. 2008). Cardiac applications currently remain limited by the fact that the mechanism of action of SCS on the heart remains unknown (Kuck et al. 2014) and by a concern that SCS might act by inhibiting the transmission of cardiac pain, thereby masking signs of impending cardiac injury (Murray et al. 2000).

Previous work conducted in the canine model supports the notion that SCS may exert myocardial effects via modulation of the intrinsic cardiac nervous system (Foreman et al. 2000; Cardinal et al. 2004; Beaumont et al. 2013). In addition to being a standard model for autonomic cardiovascular studies, canines are large laboratory animals in which it is possible to implant the same clinical‐grade neurostimulation equipment used in humans for long‐term SCS stimulation. In our hands, atrial fibrillation induced in canines by mediastinal nerve stimulation in situ was suppressed by acutely applied SCS (Cardinal et al. 2006) as well as in long‐term (3–5 week) SCS (Ardell et al. 2014). In the long‐term SCS study, we also reported preliminary data suggesting that the synaptic properties of cardiac autonomic ganglia studied in vitro were modified (Ardell et al. 2014). This is an important observation to pursue as neurotransmission in canine cardiac ganglia is attenuated in experimental heart failure (Bibevski and Dunlap 1999) and may contribute to the impaired vagal control seen in clinical heart failure (Bibevski and Dunlap 2011).

Herein, we report in vitro data obtained in experiments conducted in canines subjected to SCS extended over 5–8 weeks. Using excised right atrial ganlionated plexus (RAGP) preparations, we investigated the propositions that (1) synaptic efficacy was preferentially facilitated in long‐term SCS at high presynaptic stimulation frequency (up to 50 Hz), and (2) intrinsic neuronal membrane and action potential properties as well as muscarinic synaptic mechanisms were modified by long term‐SCS. In the canine heart, the RAGP is an epicardiac ganglionated plexus nested in a large fat pad that is readily identified at the right pulmonary vein‐right atrial junction, receiving vagal inputs via mediastinal nerves entering the epicardium and providing postganglionic innervation to the right atrial wall and sinus node region (Randall et al.^1987^; Pauza et al. 1999; Cardinal et al. 2009).

## Materials and Methods

### Ethical approval and animals

Experiments were conducted in accordance with the *Guide to the Care and Use of Experimental Animals* (Canadian Council on Animal Care, Volume 1, 2nd ed., 1993, Ottawa, Ont., Canada; www.ccac.ca/en_/standards/guidelines). These guidelines are in accord with NIH and European Union guidelines. All research was approved by the Animal Research Ethics Committee of the Sacré‐Coeur Hospital Research Center. Main comparisons were made between tissues taken from 2 groups of canines: (1) 17 animals subjected to long‐term spinal cord stimulation (long‐term SCS) continuously for 5–6 weeks (5 animals) or 8 weeks (12 animals) via a chronically implanted epidural electrode catheter, and (2) 15 control animals subjected to a sham implantation procedure in which an inactive fluid‐filled catheter was implanted. Long‐term SCS and control animals were prepared and kept simultaneously in the same housing quarters. Additionally, in a separate study, four animals were investigated following acute epidural electrode implantation and neurostimulation for 1 h (acute SCS); data from these were compared with results from four control animals subjected to a similar acute procedure but without active SCS.

### Spinal cord stimulation

Epidural catheters were implanted as reported previously (Cardinal et al. 2006; Ardell et al. 2014) in sterile conditions under isoflurane (1%) anesthesia. The animals were placed in the prone position and the epidural space of the midthoracic spinal canal was penetrated percutaneously with a Touhy needle, using fluoroscopic guidance and loss‐of‐resistance technique. An octopolar electrode (Octrode™ Model 3086; St.Jude Medical, Plano, TX) was introduced via the Touhy needle in the long‐term SCS animals, and an epidural catheter filled with contrast fluid (Arrow^®^ Flex Tip Plus^®^ Epidural Catheter; Teleflex Medical Canada, Inc., Markham, ON, Canada) was introduced in the control animals. The catheter tip was advanced to the T1 spinal level, slightly to the left of midline, and the caudal end was exteriorized and secured to the dorsal musculature with suture. In the SCS animals, the rostral and caudal poles of the electrode catheter selected for subsequent use were positioned at the T1 and T4 levels, respectively, and connected to an implantable pulse generator (IPG; EonC™ Model 3688; St.Jude Medical) that was placed in a subcutaneous pocket. In accordance with clinical use (Eliasson et al. 1996; Ekre et al. 2002), high‐frequency pulses (50 Hz, 0.2‐msec duration) were delivered at an intensity setting of 90% of motor threshold (contraction of proximal forepaw, shoulder, trunk musculature). After fixing in place the exteriorized segment of the catheter (and IPG), the surgical wounds were closed. In the long‐term SCS animals, the motor threshold was checked, after which the IPG (with receiver function) was turned off via the external programming console and remote controller. The animals instrumented for long‐term study were transferred to the recovery room. Postoperative care included analgesic (buprenorphine, 0.02 mg/kg sc at 8 h intervals for 2 days) and antibiotics (Duplocillin^®^ LA, 1 mg/10 kg sc, once a day for 3 days; Merck Animal Health – Intervet Canada Corp., Kirkland, QC, Canada). SCS was activated on the second postoperative day and maintained continuously for 5–8 weeks. Adequate IPG function was checked weekly from the pulse artifacts visible on a standard 3‐lead electrocardiogram. If needed, the motor threshold was rechecked and pulse intensity settings were adjusted with the use of the external programmer and remote controller.

### Terminal procedure and in vitro preparation

After SCS for 5–8 weeks, anesthesia was induced with Na thiopental (25 mg/kg iv), the animals were intubated and ventilation was maintained under positive pressure. A right‐sided thoracotomy was performed and the pericardium was incised to expose the heart. A bolus dose of heparin (1.5 mL, 1000 USP units/mL) was injected into the heart and the animal was killed by exsanguination as cardiac vessels were severed to remove the heart. Surgical sections were made beginning with the inferior vena cava. Special care was taken during dissection of the right pulmonary veins because of their proximity to the fat pad hosting the RAGP to avoid damaging these tissues. A segment of the right atrial wall that included the RAGP and associated myocardial and fatty tissues was quickly excised and placed in a dish containing cold (4°C) Tyrode's solution (composition in mmol/L: NaCl 128, NaHCO_3_ 20.1, NaH_2_PO_4_ 0.47, KCl 4.69, MgSO_4_ 1.18, CaCl_2_ 2.23, d‐glucose 11.1; pH 7.4) for further dissection. The myocardium surrounding the fat pad containing the ganglionated plexus was trimmed away and the remaining tissue was pinned epicardial side up to the silicone‐rubber covered bottom of a recording chamber (volume = 5 mL). This tissue was continuously superfused (5–8 mL min^−1^) by gravity from a reservoir filled with Tyrode's solution saturated with a gas mixture of 95% O_2_ and 5% CO_2_ to ensure adequate tissue oxygenation. Solution temperature was maintained at 34°C throughout the experiment by a constant‐temperature control system.

Procedures for dissection, exposure, and mechanical stabilization of canine intracardiac ganglia have been previously reported (Smith et al. 2001a,b). Briefly, the preparation was epi‐illuminated with a focal fiber‐optic light guide and viewed through a stereo microscope (model K‐401‐L; Motic Instruments Inc., Richmond, Canada). The epicardial sheath was removed and plexus nerves were identified by dissecting through the underlying fat. Ganglia were usually located at the junctions of two or more nerves or, exceptionally, attached to the side of a single nerve. An exposed ganglion was partially freed from attached connective tissue and mechanically stabilized for microelectrode impalement with the aid of a small metal platform (0.5 × 1 mm) held in a micromanipulator and inserted under the ganglion.

### Intracellular recording

Microelectrodes, made from borosilicate glass tubing (0.5 mm ID, 1.0 mm OD with internal filament; type BF‐100‐50‐10, Sutter Instruments, Novato, CA), were pulled on a Brown‐Flaming micropipette puller (Sutter Model P97) to tip diameters giving resistances in the range 20–50 MΩ when filled with 3 mol/L KCl. These electrodes were advanced with a mechanical manipulator (MX‐4; Narishige, Tokyo, Japan) into the ganglion under study to search for neuronal somata. The microelectrode was coupled to the headstage of an amplifier for intracellular recording and current injection (Neuroprobe^™^ Amplifier Model 1600; A‐M Systems, Everett, WA) operated in current clamp mode. The amplifier output was viewed on an oscilloscope and led to a data acquisition module (Cambridge Electronics Design, Cambridge, UK, Power 1401 Data Acquisition Unit) with analog‐to‐digital conversion capability (sampling rate 10 kHz). Before penetration of a ganglion, the tip potential of the electrode was nulled using the bridge controls of the intracellular amplifier while the electrode tip was in the bath. At the end of recording from each cell, the microelectrode was withdrawn from the ganglion and the null potential was checked. Transmembrane potential was taken as the difference between the potential measured in the bath and intracellular potential. Successful impalement was signaled by a sudden deflection of the electrode potential to a stable negative value. Criteria for accepting a cell were as follows: stable resting membrane potential more negative than −40 mV; action potentials (AP) with peak potential overshoot >0 V evoked reproducibly by current pulses delivered via the recording electrode; and membrane and active cell properties that were maintained consistently throughout the duration of impalement. Intracellular stimulus currents were generated using Spike 2.0 software (Cambridge Electronic Design – CED, Cambridge, UK) and applied via the microelectrode from a digital‐to‐analog converter (Power 1401 unit) connected to the intracellular amplifier. Transmembrane potentials and applied stimulus waveforms were viewed on an oscilloscope and stored for later analysis on the hard drive of a personal computer under Spike 2.0 software control. Plexus nerves that connected to ganglia in which neurons were impaled were stimulated extracellularly via fine bipolar silver wire electrodes coupled to a constant current or voltage stimulus isolation unit (model 2200; A‐M Systems). This unit was driven by rectangular pulses generated by the Spike 2.0 software.

### In vitro study protocol

#### Action potential properties

**Figure 1:**
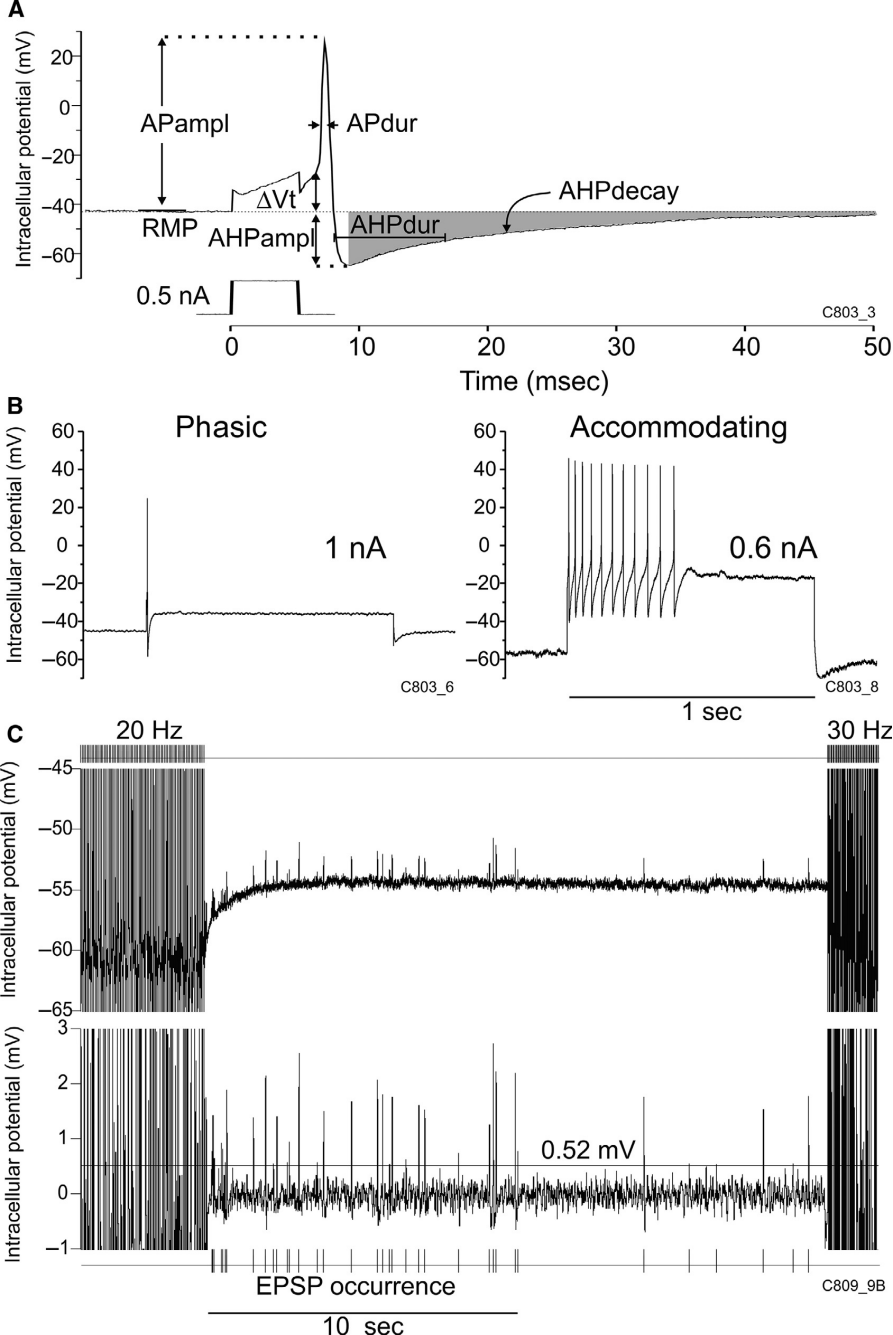
(A) Measurement of action potential (AP) and afterhyperpolarization (AHP) properties illustrated in an AP elicited by intracellular injection of a current pulse (0.5 nA, 5 msec): resting membrane potential (RMP), voltage displacement from RMP to AP threshold (Δ*V*t), AP and AHP amplitude (APampl, AHPampl) and duration (APdur, AHPdur); the time course of AHP decay was measured as the surface area between the AHP voltage curve and RMP (gray shading) over a specified time interval (here, from peak AHPampl to 250 msec). In this panel and in subsequent examples of individual neurones, the experiment and cell identification numbers are indicated in the lower right hand corner. (B) Classification of neurones as phasic (left) or accommodating (right) on the basis of AP firing limited to, or extending beyond the first 100 msec of a 1‐sec intracellular depolarizing pulse, respectively. Left hand panel: typically, a single AP was elicited in a phasic neurone at maximal current of 1 nA, whereas 11 APs spanning a 460 msec interval were elicited at 0.6 nA in an accommodating cell. (C) Example of presynaptic nerve stimulation in which successive trains of stimuli were applied at increasing frequency (here: 20 and 30 Hz, with a 20‐sec interval between trains) while recording intracellularly from a postsynaptic neurone. Upper tracing: original intracellular recording; lower: magnified tracing with DC removed and median ±5 msec filtering to determine the number and amplitude of excitatory postsynaptic potentials (EPSPs) following the stimulus train. EPSP numbers were counted as the number of deflections that exceeded a threshold of 4SD of the baseline noise around the RMP in any given neurone (here: horizontal line drawn at 0.52 mV).

Once resting membrane potential (RMP) of an impaled neurone had stabilized, 5 msec depolarizing current pulses were applied intracellularly at 1 sec intervals with increasing current (0.1–1 nA in 0.1 nA steps) until threshold for eliciting an AP (Fig. 1A). Analysis of AP properties consisted of evaluating: voltage displacement from RMP to action potential threshold (ΔVt in mV), AP amplitude (AP ampl in mV), AP duration at 50% repolarization (AP dur in msec), afterhyperpolarization amplitude (AHP ampl in mV), and AHP duration at 50% of decay back to RMP (AHPdur in msec). The time course of AHP decay was estimated as the surface area (*μ*V msec) between the AHP voltage curve and a line representing the level of pre‐AP resting membrane potential (Fig. 1A: gray shading) over a specified interval (typically, 20–250 msec from stimulus initiation). Increases and decreases in this measure corresponded to prolongation and acceleration, respectively, of the time course of AHP decay, providing a corroborative measure analogous to AHP duration in the absence of change in AHP duration. This approach also allowed us to assess the AHP time course in the last AP response to a presynaptic nerve stimulation train (see below) avoiding any interference from superimposed EPSPs in the early portion of the AHP of such responses (early 50 msec excluded).

#### Membrane input resistance

Hyperpolarizing current pulses (1 sec duration) of increasing intensity (−0.1 to −0.6 nA in 0.1 nA steps) were injected intracellularly and the resulting voltage displacements (measured at 800 msec from pulse initiation) were plotted as a function of current intensity. The membrane input resistance (MΩ) was determined from the slope of the current–voltage relationship.

#### Repetitive firing properties and membrane excitability

These were evaluated by applying 1 sec‐depolarizing current pulses intracellularly (Fig. 1B) at 5 sec intervals with increasing current (0.1–1 nA in 0.1 nA steps). Threshold current (*I*_t_) was taken as the lowest current intensity required to elicit one AP. In accordance with previous studies conducted in canine intrinsic cardiac ganglia (Smith et al. 2001a,b; Xi et al. 1994), neurones were classified as phasic (Fig. 1B, left hand panel) or accommodating (right hand panel) depending on whether APs continued to occur beyond the first 100 msec of the stimulus pulse.

#### Membrane responses to presynaptic nerve input stimulation

Single or repetitive stimulus pulses (0.5 msec duration, 100 *μ*A–5 mA) were applied extracellularly via silver wire electrodes on one or more nerves connected to the ganglion containing an impaled neuron. Single‐pulse stimuli were applied at an intensity that elicited an excitatory postsynaptic potential (EPSP) in the impaled soma, as expected for orthodromic synaptic transmission. Graded increases in stimulation intensity then resulted in proportionally graded increases in the amplitude of the evoked EPSP which, upon reaching threshold for a regenerative response, elicited an AP. To test the capability of synaptic connections to transfer information with time (synaptic efficacy), the plexus nerves were stimulated at twice threshold intensity for AP generation with trains of pulses applied successively (at 20 sec intervals) at increasing frequency (5‐pulse trains at 0.2 and 0.5 Hz, 10 pulses at 1 and 2 Hz, 25 pulses at 5 Hz, 50 pulses at 10 Hz, and 100 pulse trains at 20–50 Hz: Fig. 1C).

In addition to EPSPs evoked by nerve stimulation, EPSPSs also occurred spontaneously between stimulus trains. Spontaneous occurrence of EPSPs in canine intracardiac ganglia was previously reported by others (Xi et al. 1991). In this study, the numbers of spontaneously occurring EPSPs were counted over the 20‐sec periods between nerve stimulation trains as illustrated in Fig. 1C (see fig. legend for details).

### Pharmacological agents

Atropine sulfate and XE991 (10,10‐bis(4‐pyridinylmethyl)‐9(10H)‐anthracenone) were purchased from (Sigma‐Aldrich Canada, Oakville, Ont., Canada). These agents were freshly dissolved in small volumes of Tyrode's solution on the day of the experiment. Atropine and XE991 were introduced in the superfusion solution to achieve bath concentrations of 10 *μ*mol/L (Smith et al. 2001b) and 3 *μ*mol/L (Zaczek et al. 1998), respectively. Measurements were made after 5‐min exposure to atropine, and between 5 to 10 min of exposure to XE991.

### Signal processing and data analysis

Stored transmembrane potentials and applied stimulus waveforms were retrieved for analysis using custom programs written within the Spike 2 software suite using Cambridge Electronic Design proprietary script language. Data were transferred to spreadsheets and statistical analyses were performed using SPSS Statistics for Windows, Version 22.0, software (IBM Corp., Armonk, NY). Data on membrane properties and regenerative responses to intracellular current injection (presented as mean ± SD) were compared between the long‐term SCS and control groups by multivariate analysis of variance (MANOVA) for each neurone type (phasic, accommodating). As indicated above, classification as phasic or accommodating depended on the individual neurones’ firing properties during intracellularly applied 1 sec‐depolarizing current pulses. The frequency‐dependent following capabilities of postsynaptic AP responses (synaptic efficacy) were analyzed by ANOVA for repeated measures (effect of stimulus frequency) and between group comparisons (long‐term SCS vs. control). In addition, comparisons were made between trials conducted in basal states (baseline) and repeated under atropine superfusion (data from the control and experimental groups were combined for these comparisons). Likewise, ANOVA for repeated measures (frequency, atropine) and between group comparisons were used to analyze posttrain EPSP numbers and AHP decay surface area. Differences were considered as statistically significant when *P* ≤ 0.05.

## Results

### Membrane properties and evoked regenerative responses

Intracellular recordings were obtained from 124 neurones sampled from RAGP tissues isolated from the hearts of 15 sham animals and from 150 neurones sampled from 17 animals with long‐term SCS (yield ~8 cells/specimen). Phasic neurones typically discharged a single AP (occasionally 2–4) during the first 100 msec of intracellular depolarizing pulses applied for 1 sec. There was no difference in the discharge patterns of phasic neurons sampled from control and long‐term SCS animals. The number of APs discharged by accommodating neurones during 1 sec stimulus pulses varied with the intensity of the stimulus current and there was no difference between accommodating neurones from control and long‐term SCS animals in the increment of AP frequency as current was increased. Also, about twice as many phasic than accommodating neurones were sampled from control and long‐term SCS hearts (Table 1).

**Table 1:** Membrane properties and regenerative responses to intracellular current injection

Group cell type (*n*)	Rin (MΩ)	*I*_t_ (nA)	RMP (mV)	Δ*V*_t_ (mV)	AP ampl (mV)	AP during (msec)	AHP ampl (mV)	AHP during (msec)	AHP decay surface area (mV × msec)
Control phasic (80)	56 ± 50	0.40 ± 0.24	−50 ± 9	20 ± 5	59 ± 9	1.5 ± 0.6	13 ± 3	28 ± 24	423 ± 385
Control accom (44)	97 ± 58	0.19 ± 0.07	−51 ± 8	20 ± 5	58 ± 12	1.6 ± 0.4	13 ± 3	33 ± 20	416 ± 364
SCS phasic (103)	37 ± 32^a^	0.48 ± 0.26^a^	−48 ± 8	21 ± 5	62 ± 12	1.2 ± 0.4^a^	14 ± 3	22 ± 20^a^	310 ± 307^a^
SCS accom (47)	86 ± 46	0.19 ± 0.07	−50 ± 7	21 ± 6	61 ± 12	1.6 ± 0.6	14 ± 4	32 ± 20	419 ± 335

Data are expressed as mean ± SD.

Accom, accommodating neurone; Rin, whole‐cell input resistance; *I*_t_, threshold current; RMP, resting membrane potential; Δ*V*_t_, voltage displacement from RMP to action potential threshold; AP ampl: action potential amplitude; AP dur, action potential duration; AHP ampl, afterhyperpolarization amplitude; SCS, spinal cord stimulation; AHP dur, AHP duration at 50% decay to RMP; nA, nanoampere; MΩ, megohm; mV, millivolt; msec: millisecond.

a *P *<* *0.05 comparing SCS versus Control.

**Figure 2:**
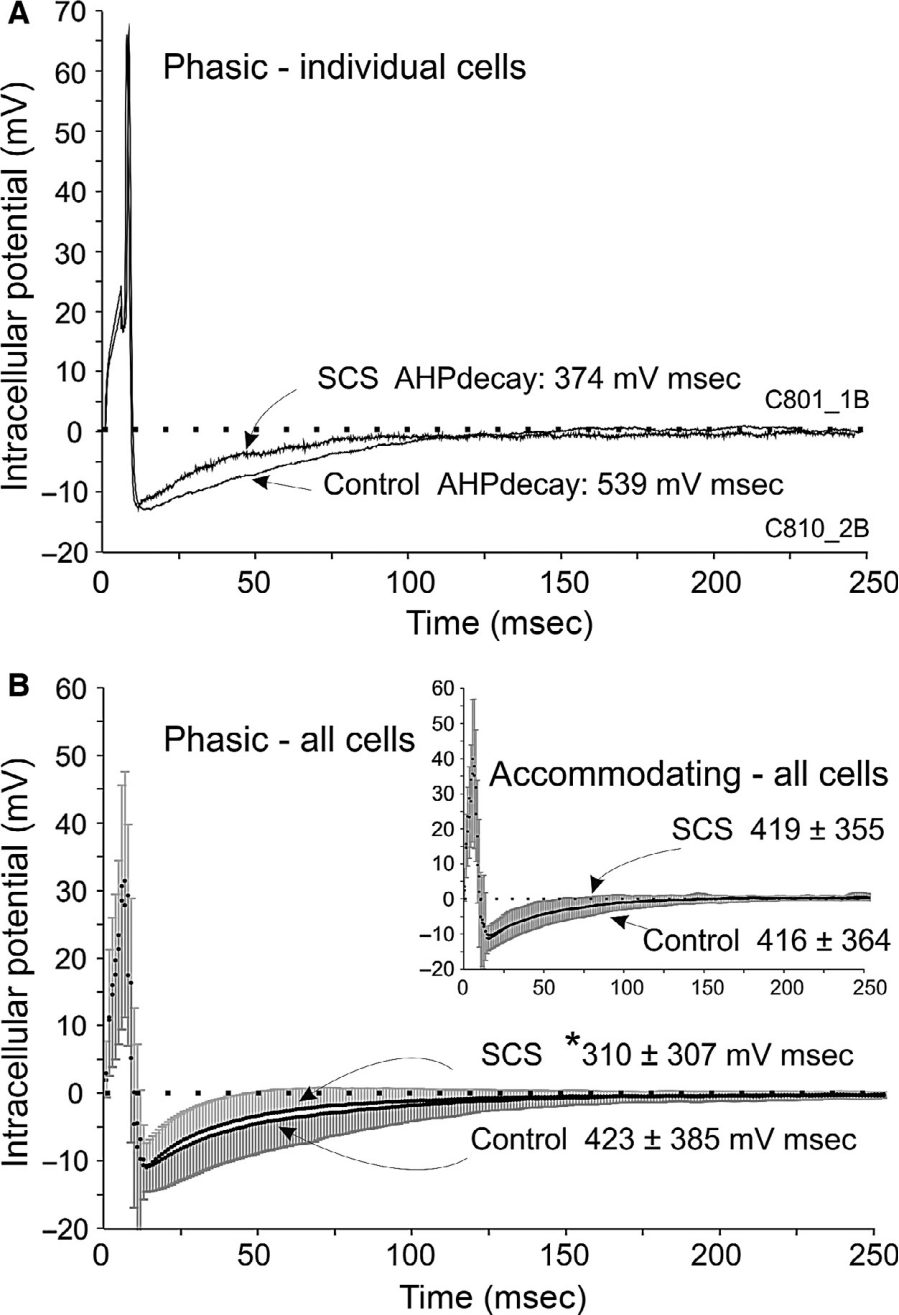
Modification of the time course of AHP decay in phasic neurones from the long‐term SCS group. (A) Superimposed action potential (AP) recordings from individual phasic neurones in preparations from long‐term SCS (upper trace) and control (lower trace); APs evoked by intracellular pulse stimulation are shown superimposed, with their respective resting membrane potentials normalized to 0 potential on the ordinate axis (dotted horizontal line). Note that the surface area of AHP decay was smaller in the SCS than in the control recording. AHP durations differed accordingly (SCS: AHPdur = 22 msec, compared with control: 32 msec). (B) Main curves – phasic neurones: summated AP recordings from long‐term SCS (upper trace: mean of *n* = 100 cells, upward SD) and superimposed summated recordings from controls (lower trace: mean of *n* = 76 cells, downward SD). The time course of AHP decay surface area (measured up to 250 msec) was significantly smaller in the long‐term SCS than in controls. Inset‐accommodating neurones had similar AHP decay surface area values in SCS and control; same presentation as for main curves.

Whole‐cell membrane input resistance of phasic neurones was significantly lower in long‐term SCS than control neurones (Table 1: Rin, *P* < 0.05); accordingly, threshold currents (*I*_t_) tended to be higher in the phasic neurones of the long‐term SCS group. There were no differences between groups in RMP, voltage displacement to threshold potential (Δ*V*_t_) or action potential amplitude (AP ampl). Action potential duration (AP dur) was significantly shorter in phasic neurones of the long‐term SCS group than control. There was no statistically significant difference in afterhyperpolarization potential amplitude (AHP ampl) between groups. The time course of AHP decay was significantly accelerated in phasic neurones from long‐term SCS animals in comparison with control phasic neurones (Table 1: AHP decay surface area) as illustrated in APs from individual cells (Fig. 2A) and averaged APs from all cells (Fig. 2B: main curves). This corresponded to a tendency for shorter AHP duration at 50% decay among phasic neurones (Table 1, AHP dur). However, the time course of AHP decay values were similar in accommodating neurons from control and long‐term SCS hearts (Fig. 2B: inset curves).

### Differential responses to synaptic activation in long‐term SCS versus control

**Figure 3:**
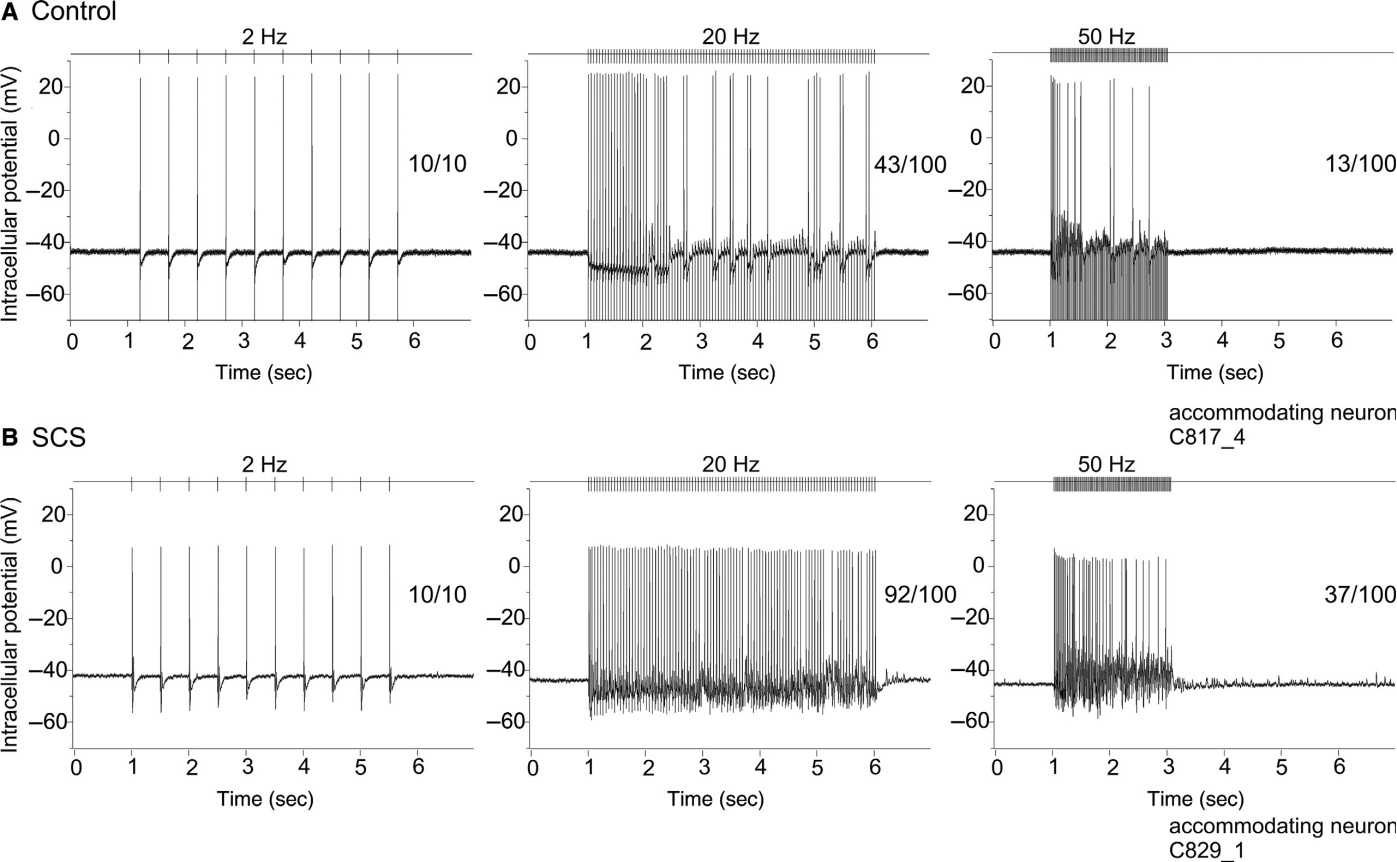
Representative examples of postsynaptic responses to repetitive presynaptic nerve stimulation in control and long‐term SCS. (A) Intracellular recording from a representative accommodating neurone of the control group illustrates that one‐to‐one orthodromic transmission (presynaptic pulse number / postsynaptic action potential number) occurred at low repetitive stimulation frequency (10/10 at 2 Hz) whereas synaptic efficacy decreased at high nerve stimulation frequencies (43/100 at 20 Hz and 13/100 at 50 Hz). (B) In a representative example from the long‐term SCS group, synaptic efficacy was more robust than control at high presynaptic nerve stimulation frequencies: 92/100 at 20 Hz, and 37/100 at 50 Hz.

**Figure 4:**
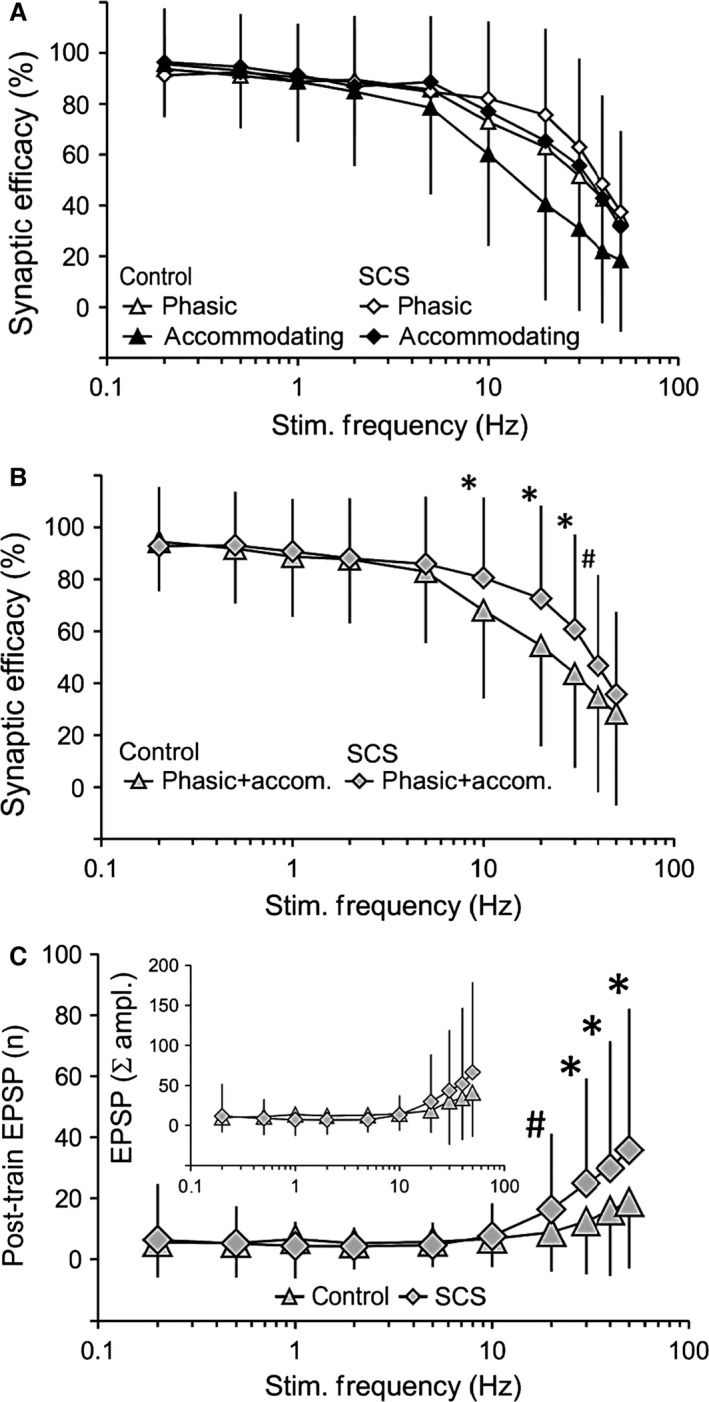
Differential improvement of synaptic efficacy at high presynaptic nerve stimulation frequencies and increased occurrence of spontaneous EPSPs in long‐term SCS versus control. (A) Cumulative data (*n* = 52 control and 67 long‐term SCS neurones) illustrating synaptic efficacy (ordinate) measured as % of presynaptically applied pulses that elicited postsynaptic action potentials, as a function of presynaptic nerve stimulation frequency plotted on logarithmic scale (abscissa). Synaptic efficacy shown for phasic and accommodating neurones in control and long‐term SCS was significantly reduced at increasing frequency, with the greatest reduction occurring among accommodating neurones of the control group. (B) Combining cell types (phasic+accom.) in each group, synaptic efficacy was significantly more robust at high nerve stimulation frequency in long‐term SCS than control. (C) The number (*n*: main curves) and summed amplitude (inset) of excitatory postsynaptic potentials (EPSPs) occurring spontaneously following presynaptic nerve stimulation trains (post‐train) increased at stimulus frequency >20 Hz. Post‐train EPSP numbers were significantly higher in long‐term SCS than control at frequencies >20 Hz. For panels (A, B and C): data are mean ± SD, *P <* *0.05, ^#^marginally significant, *P *=* *0.06.

When plexus nerves were stimulated with increasing frequency (0.2–50 Hz), changes in synaptic efficacy were expressed as the numbers of action potentials evoked by each stimulus train expressed as a fraction (%) of the number of pulses delivered in the train. This is illustrated in a record from a representative accommodating neurone taken from the control group (Fig. 3A). In this example, one‐to‐one orthodromic transmission occurred at low stimulation frequencies (10/10 at 2 Hz), whereas this relationship was reduced at higher stimulation frequencies (Fig. 3A: 43/100 at 20 Hz and 13/100 at 50 Hz). In a representative example from the long‐term SCS group (Fig. 3B), synaptic efficacy was more robust than in the neurone from the control group at high presynaptic nerve stimulation frequencies (92/100 at 20 Hz and 37/100 at 50 Hz). This difference was substantiated in cumulative data (Fig. 4A, *n* = 52 control and 67 long‐term SCS neurones). There was thus a significant overall inverse dependence of postsynaptic responses on presynaptic stimulation frequency (*P* < 0.001), the synaptic efficacy being more robust to increments in presynaptic stimulation frequency among phasic than accommodating neurones (frequency × cell type interaction: *P* = 0.002). Importantly, the decrease in synaptic efficacy was significantly greater among cells from the control group in comparison with cells from the long‐term SCS group at 10 Hz (*P* = 0.028), 20 Hz (*P* = 0.006), 30 Hz (*P* = 0.009) and marginally at 40 Hz (*P* = 0.06) as illustrated in Figure 4B. This accorded with the representative examples shown in Figure 3.

When the spontaneously occurring EPSPs were counted during the 20‐sec interval between the nerve stimulation trains, EPSP count (Fig. 4C: main curves) and summated EPSP amplitudes (inset) increased following presynaptic nerve stimulation at frequencies beyond 10 Hz. Furthermore, the increase in number of EPSPs was significantly greater in preparations from the long‐term SCS group than control.

### Inhibition of synaptic transmission by atropine

**Figure 5:**
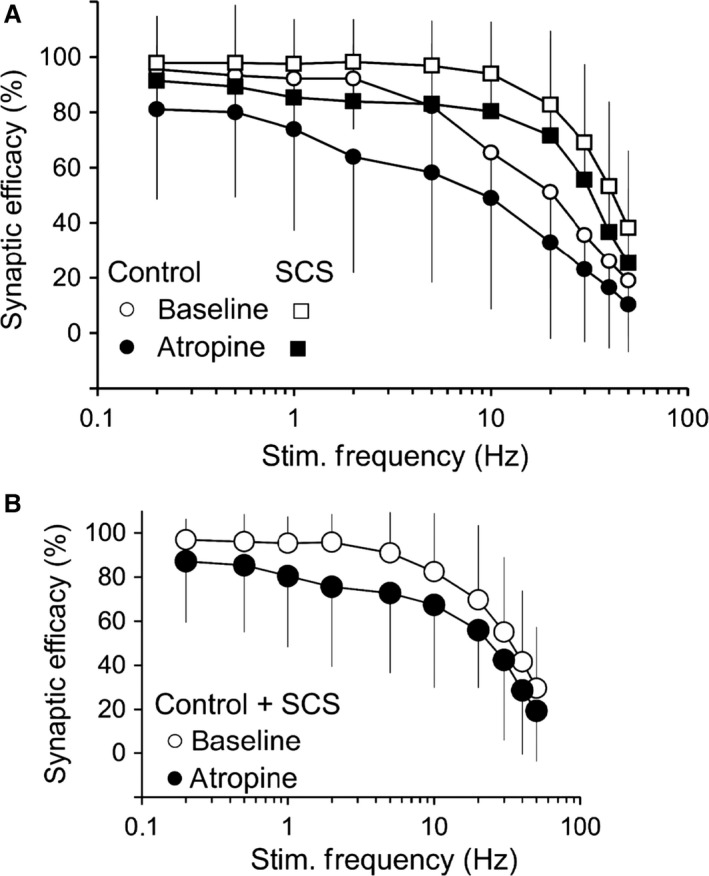
Inhibition of synaptic transmission by atropine. (A) Synaptic efficacy (ordinate) was measured as % of presynaptically applied pulses that elicited a postsynaptic action potential, as a function of presynaptic nerve stimulation frequency plotted on a logarithmic scale (abscissa). Synaptic efficacy is shown for neurones (cell types combined) in the control group (*n* = 18, circles) and long‐term SCS group (*n* = 27, squares) in basal states (baseline: clear symbols) and during exposure to atropine 10 *μ*mol/L (dark symbols). B. The curves illustrate that atropine produced a statistically significant (*P* < 0.001) reduction in synaptic efficacy in the two groups combined (Control + long‐term SCS); clear circles: baseline; dark circles: atropine. Panels A and B: data are mean ± SD.

In a subgroup of the preparations reported above, evaluation of synaptic transmission in basal states was repeated during exposure to atropine (Fig. 5A). Synaptic efficacy was significantly reduced in the presence of atropine (atropine effect, *P* < 0.001); this reduction occurred equally in preparations from both the control and long‐term SCS groups and at all frequencies (no significant atropine × group, or atropine×frequency interactions). Figure 5B illustrates the significant atropine effect in the two groups combined (*P* < 0.001).

### Differential effects of atropine on the time course of AHP decay in responses to presynaptic nerve stimulation

**Figure 6:**
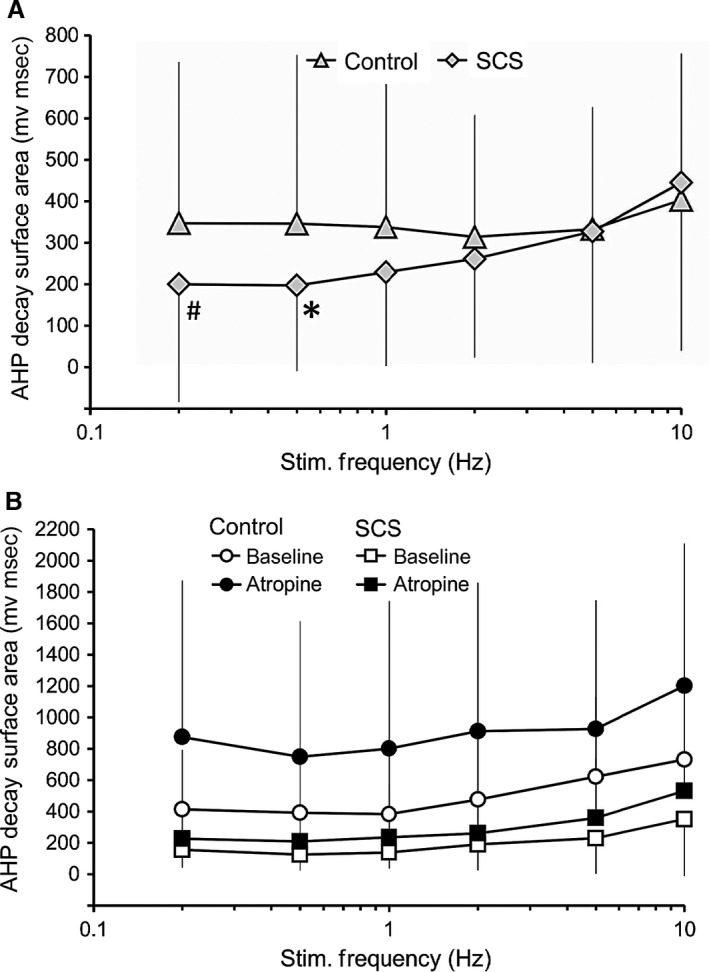
Differential effects of atropine on the time course of AHP decay following presynpatic nerve stimulation in control versus long‐term SCS. In both panels, AHP decay surface areas were estimated from the AHP following the last AP of each stimulus train. (A) The time course of AHP decay was faster at low presynaptic nerve stimulation frequencies in preparations from long‐term SCS (*n* = 47) compared to control (*n* = 24) (*P < 0.05, ^#^marginally significant, *P* = 0.06). (B). In a subgroup of preparations that was exposed to atropine (control, *n* = 19; SCS,* n* = 6), atropine (10 *μ*mol/L) induced a prolongation of the time course of AHP decay across all frequencies in neurones from control but not among those from long‐term SCS. Data are mean ± SD.

The relationship between the time course of AHP and nerve stimulation frequency was significantly different between preparations from the control and long‐term SCS groups (frequency × group interaction, *P* < 0.05), with a significantly faster time course of AHP decay at lower stimulation frequency in long‐term SCS (Fig. 6A). Moreover, across all nerve stimulation frequencies, there was a significant prolongation by atropine of the time course of AHP decay in neurones from control but not in those from long‐term SCS (Fig. 6B).

### Effects of XE991 on neuronal excitability

**Figure 7:**
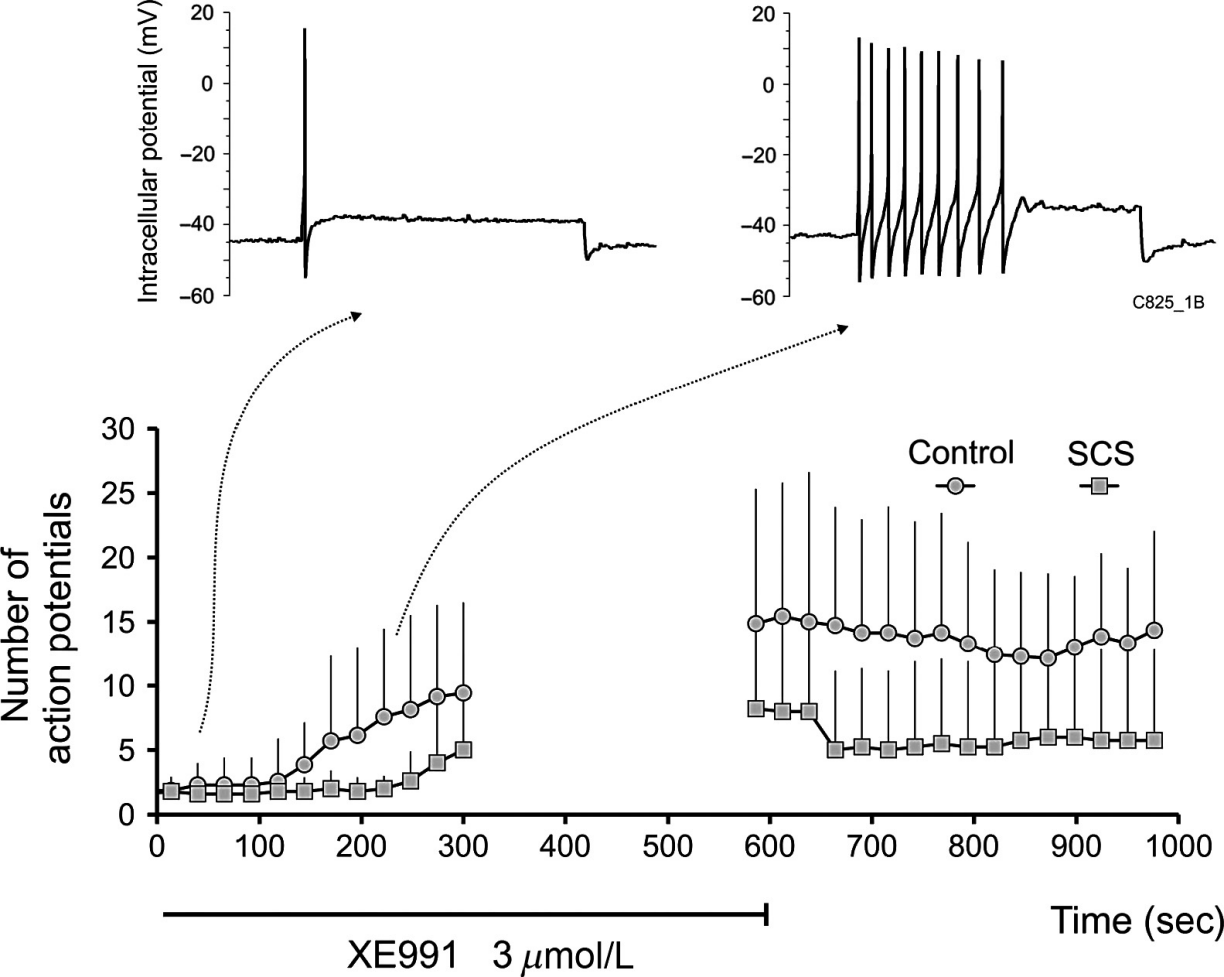
Effects of XE991 on neuronal excitability in control and long‐term SCS. Number of action potentials (AP) induced by 1‐sec intracellular current pulses injected at 26 sec intervals during XE991 (3 *μ*mol/L) superfusion increased from a single AP in most cells in basal states to a mean of 17 ± 11 APs in neurones from the control group (*n* = 7) and to 9 ± 8 APs in long‐term SCS (*n* = 5) at peak XE991 effect. Upper traces show representative examples from the control group at early and later times during XE991 superfusion. Excitability measurements were interrupted between 300 and 580 sec.

When 1‐sec intracellular current pulses were injected at 26 sec intervals during XE991 exposure (Fig. 7), AP firing increased over a 5–10 min exposure from a single AP in most cells to a mean of 17 ± 11 APs in neurones from control (*n* = 7), and to 9 ± 8 APs in long‐term SCS (*n* = 5). There was a statistically significant XE991 exposure time effect (within), but no statistically significant between group effect (long‐term SCS vs. control).

### Acute (1‐h) SCS

Comparing animals subjected to SCS for only 1 h with matching control animals subjected to an acute sham procedure, no differences were identified in the intrinsic neuronal properties except for a significantly longer AHPdur and a tendency for a prolonged time course of AHP decay among the accommodating neurones of the acute SCS group (data not shown). Synaptic efficacy curves showed a statistically significant effect of nerve stimulation frequency, but without any significant difference in this property between groups.

## Discussion

The major finding of this study was that long‐term SCS has the capability of modifying the neuronal intrinsic membrane properties and neurotransmission within the intracardiac nervous system. First, in phasic neurones from the long‐term SCS group, there was a significant reduction in whole‐cell input resistance, shortening of the action potential duration and acceleration of the time course of AHP decay. Second, synaptic transmission at high presynaptic nerve stimulation frequencies (≥10 Hz) was significantly more robust in long‐term SCS than in preparations from the control group. This was accompanied by significantly higher numbers of spontaneous EPSPs (and their summated amplitudes) following trains of high‐frequency nerve stimulation in the long‐term SCS group. Third, synaptic efficacy was significantly decreased by pharmacological manipulation of muscarinic receptors with atropine in both the control and long‐term SCS groups. Moreover, the time course of AHP decay in the last of a train of APs evoked by presynaptic nerve stimulation was significantly prolonged in the presence of atropine among the neurones from the control group but not among the ones from the long‐term SCS group.

### SCS modulation of neuronal intrinsic membrane properties

Several K^+^ (or cation‐selective) currents are involved in shaping the action potential configuration and course of AHP decay, as well as setting the neuronal membrane properties and active discharge behavior in response to a depolarizing current pulse and to synaptic inputs in mammalian intrinsic cardiac neurones (Xi‐Moy and Dun 1995; reviewed by Adams and Cuevas 2004).

Whole‐cell membrane input resistance was significantly reduced, AP and AHP durations were significantly shortened, and the time course of AHP decay was significantly accelerated in phasic neurones from the long‐term SCS group in comparison with neurones from the control group (summarized in Table 1, Fig. 2). The interpretation of these findings calls for the consideration of several ionic conductances in relation to their possible contributions to such events occurring at different levels of membrane potential and at different times, in keeping with the currents’ known voltage‐dependent and kinetic characteristics. We surmise that such changes identified in long‐term SCS phasic neurones might be explained–on the basis of the relevant literature (reviewed by Adams and Cuevas 2004) ‐, by increased K^+^ conductances during AP repolarization (*e.g*. delayed outward rectifying K^+^ current) and at hyperpolarized membrane potentials at which whole‐cell input membrane resistance was measured in this study (e.g. inwardly rectifying K^+^ current).

Concerning the K^+^ currents possibly underlying the AHP, it is interesting to note that Xi et al. (1994) reported that in similar canine intrinsic cardiac neurones, AHP duration was significantly shortened (from a mean of 55.5 msec to 22.9 msec) during superfusion with a low Ca^2+^/high Mg^2+^ solution, suggesting the involvement of Ca^2+^‐sensitive K^+^ currents. Muscarine‐sensitive K^+^ conductances may also modulate the amplitude and time course of the AHP (Allen and Burnstock 1990). It is noteworthy that a muscarine‐sensitive current (presumed to be *I*_M_) has been reported to be expressed to a relatively greater extent in phasic neurones of guinea pig sympathetic ganglia, whereas transient outward currents resembling A‐current (*I*_A_) were evoked by depolarization from resting membrane potential in tonic neurones (and, by extension, accommodating neurones) (Cassell et al. 1986). In canine intrinsic cardiac neurones, both voltage‐sensitive Na^+^ and Ca^2+^ channels were shown to contribute to the generation of APs by a short duration intracellular current pulse (blocked by combined tetrodotoxine and low Ca^2+^/high Mg^2+^) but sensitivity to tetrodotoxine was 10‐fold higher in tonic cells than in phasic cells (Xi et al. 1994). Such currents may have been differentially altered in long‐term SCS.

A well‐documented effect of inhibiting I_M_ and other K^+^ conductances evoked by depolarization from resting membrane potential is an increase in neuronal excitability (Brown and Adams 1980; Cassell and McLachlan 1987; Allen and Burnstock 1990; Cuevas et al. 1997). In this study, the relatively selective blockade of I_M_ with XE991 (Zaczek et al. 1998) induced phasic and short‐accommodating neurones from both the control and SCS groups to discharge significantly more APs during 1‐sec intracellular depolarizing pulses (Fig. 7) but without statistically significant between group effect, with a limited number of cells in each group.

### SCS modulation of intracardiac ganglionic neurotransmission

Synaptic transmission was significantly reduced during high‐frequency repetitive activation in the preparations studied herein. A major finding was that synaptic efficacy was significantly facilitated at high presynaptic nerve stimulation frequencies (≥10 Hz) in long‐term SCS in comparison with preparations from control animals. In principle, facilitation could have occurred as a result of modulation of presynaptic or postsynaptic mechanisms, or both. In intracardiac and other autonomic ganglia, the amplitude of EPSPs evoked by orthodromic stimulation diminishes as frequency increases (Seabrook and Adams 1989; Seabrook et al. 1990). This effect is consistent with a reduction in the amount of acetylcholine released per AP from presynaptic nerve terminals and may be caused, in part, by activation of muscarinic receptors that inhibit Ca^2+^ entry into the nerve terminal, resulting in synaptic rundown (Brown 2010). It is possible that – in long‐term SCS – the density of presynaptic muscarinic receptors may have been reduced, thus contributing to strengthening synaptic efficacy.

On the postsynaptic membrane, functional downregulation of any of the several K^+^ conductances possibly involved in opposing membrane depolarization (EPSP) might explain a significantly greater synaptic efficacy at rapid presynaptic stimulation frequency in long‐term SCS than in neurones from control animals (Figs. 3 and 4). If muscarine‐sensitive K^+^ conductances were involved, blockade of muscarinic receptors with atropine (which would restore the inhibitory role of K^+^ conductances on excitability) should suppress postsynaptic firing, as was indeed observed in neurones from both the control and long‐term SCS animals (Fig. 5). Interestingly, exposure to atropine mediated a prolongation of the time course of AHP decay that followed the train of APs evoked by nerve stimulation in neurones from control but not from long‐term SCS animals (Fig. 6).

Given that synaptic efficacy – studied in fully polarized cells – was found to be more robust in long‐term SCS whereas excitability was similar to sham in responses to 1‐sec depolarization steps, the data are consistent with functional downregulation of a current activated in the negative transmembrane potential range at which EPSPs occur. Downregulation of I_M_ (Brown and Adams 1980; Xi‐Moy and Dun 1995; Cuevas et al. 1997) or of any of the several different conductances shown to be affected (inhibited) by muscarinic stimulation in rat or guinea‐pig sympathetic neurones should be considered (Cassell and McLachlan 1987; Cuevas and Adams 1997). A limitation in discussing, in mechanistic terms, the data reported herein is that most of the data available regarding the electrophysiological properties of neurones in mammalian autonomic ganglia were derived from rat and guinea‐pig models (reviewed by Adams and Cuevas 2004), whereas fewer studies have been conducted in canine intrinsic cardiac neurones (Xi et al. 1991, 1994; Xi‐Moy et al. 1993; Smith et al. 2001a,b).

During the interval between high‐frequency presynaptic stimulus trains (illustrated in Fig. 1C), spontaneous EPSPs occurred in both phasic and accommodating neurones and in significantly higher numbers in long‐term SCS neurones than in those from control animals (Fig. 4C). Such potentials have been reported previously in intrinsic cardiac neurones sampled in in vitro preparations (Xi et al. 1991). Whereas their origin is uncertain, they may represent ongoing activity possibly arising from putative afferent neurones and interposed local circuit neurones existing in the cardiac ganglionated plexuses (Thompson et al. 2000). Functionally downregulated K^+^ conductances in the range of membrane potentials at which EPSPs occur could have potentiated such spontaneous postsynaptic events in long‐term SCS neurones, as we have proposed for neutrally evoked postsynaptic events.

Intrinsic membrane property modifications in basal states (shortening of AP and AHP durations) were identified in long‐term SCS neurones among the phasic ones only, whereas facilitation of synaptic efficacy was a group effect (long‐term SCS vs. control). Therefore, it may be concluded that the mechanisms underlying synaptic facilitation may be different between the phasic and accommodating neurones of the long‐term SCS group and that the relationship between such facilitation and modification of their intrinsic membrane properties remains to be elucidated in future work.

### Relation to previous work

Data reported herein imply that the changes observed in long‐term SCS preparations resulted from electrophysiological remodeling over the 5–8‐week period after activation of the neurostimulator. However, it should be considered whether some of the changes detected in the long‐term SCS group may have resulted from rapid adaptations to acute SCS in situ (Cardinal et al. 2004, 2006) that may have then persisted in the in vitro study since the neurostimulator was turned off just prior to animal sacrifice and heart removal for tissue collection. To control for this possibility, we studied neurones from animals in which SCS was performed for 1 h and from matching control animals that had been subjected to a 1‐h sham procedure without active SCS. We found no differences in neuronal properties or synaptic transmission in tissues taken from such acute SCS and control animals, so we conclude that the changes observed in samples from long‐term SCS animals were in fact the result of time‐dependent remodeling within the intrinsic cardiac nervous system.

Herein, we have confirmed and extended previously reported preliminary in vitro findings in long term‐SCS (Ardell et al. 2014). Analysis of data from a larger number of animals and cell numbers in this study has shown significant long‐term SCS‐related changes in membrane and active properties of phasic neurones, specifically highlighting modification of whole‐cell input resistance, AP duration and AHP duration among phasic neurones. In this study, we investigated animals that had been subjected to SCS for up to 8 weeks, as opposed to a maximum of 5 week in the previous study. This extended time frame may also have contributed to reveal changes in the electrophysiology of intrinsic cardiac neurones that were not apparent or did not exceed threshold for statistical significance in the previous study. Here, we also extended the examination of postsynaptic responses during presynaptic nerve stimulation to explore frequencies up to 50 Hz (the previous study was limited to 20 Hz maximum). This has confirmed that significant changes in intracardiac ganglionic neurotransmission were promoted by long‐term SCS, and has provided a much clearer picture of the nature and extent of these changes. In this respect an important finding was that the robustness of synaptic transmission at high presynaptic stimulation frequencies was greater in long‐term SCS than control. It is noteworthy that such differences in synaptic efficacy were consistently identified in the several experimental series that comprised this study, carried out at different times over a 2‐year period, thus supporting the reproducibility of the reported long‐term SCS effects.

### Perspective

There is general agreement that the intrinsic cardiac ganglia may play a role in “gating” parasympathetic (vagal) influences onto the heart as well as processing local inputs from the heart (Armour and Kember 2004). However, the relative roles of phasic versus accommodating (or tonic) neurones remain unclear, some authors ascribing the “gating” functionality of vagal inputs to the former (Xi et al. 1994; Smith 1999; working with canine and porcine excised RAGP preparations, respectively) whereas the latter have been favored by others (McAllen et al. 2011; in a working heart‐brainstem rat preparation). In spite of such differences, it can readily be concluded that remodeling of the membrane properties and synaptic behavior of intrinsic cardiac neurones by long‐term SCS occur directly at the input portal of the cardiac nervous system and modify vagal influences onto the heart. Accordingly, in our hands and others’, SCS acutely applied in anesthetized canines caused a reduction in spontaneous heart rate (Issa et al. 2005; Cardinal et al. 2006) as well as potentiation of bradycardia and atrial repolarization changes evoked by electrical stimulation of the right cervical vagus nerve (Jacques et al. 2011). Moreover, chronic treatment with SCS (2 h, *t.i.d*.) significantly reduced ambulatory heart rate in conscious canines at 2, 5, and 10 weeks (Lopshire et al. 2009).

Vagus nerve influences onto the heart are impaired in heart failure, as evidenced by decreased heart rate variability in the high‐frequency power spectrum (reviewed by Bibevski and Dunlap 2011), and vagal stimulation is under investigation for improvement of cardiac status (Buckley et al. 2015; Tse et al. 2015). The improvement of neurotransmission at rapid presynaptic drive in long‐term SCS could provide a physiologically modulated mechanism to facilitate vagal inputs to the heart. The mechanism whereby high‐frequency submotor threshold SCS may cause remodeling of the membrane and synaptic properties of autonomic neurones remains unknown. Experiments in canines show that its anti‐ischemic (Foreman et al. 2000) and antiarrhythmic effects (Cardinal et al. 2006) depend on intact cardiac nerve connections via the stellate ganglia. By analogy with the beneficial effects of SCS applied to lumbar SC segments in peripheral lower limb ischemic disease (Wu et al. 2008), it can be speculated that SCS might cause the release of neuromodulatory (peptide?) substance(s) that might affect intrinsic cardiac neural function acutely (Cardinal et al. 2006) and induce synaptic remodeling in the long‐term (Ardell et al. 2014 and this report).

## Conflict of Interest

None declare.
